# Anti-masculinization induced by aromatase inhibitors in adult female zebrafish

**DOI:** 10.1186/s12864-019-6437-z

**Published:** 2020-01-07

**Authors:** Lu Chen, Li Wang, Qiwei Cheng, Yi-Xuan Tu, Zhuang Yang, Run-Ze Li, Zhi-Hui Luo, Zhen-Xia Chen

**Affiliations:** 1 Hubei Key Laboratory of Agricultural Bioinformatics, College of Life Science and Technology, Huazhong Agricultural University, Wuhan, Hubei 430070 People’s Republic of China; 2 College of Biomedicine and Health, Huazhong Agricultural University, Wuhan, Hubei 430070 People’s Republic of China

**Keywords:** Sex differentiation, Zebrafish, RNA-Seq, Adult, Aromatase inhibitor, Sex reversal

## Abstract

**Background:**

Early sex differentiation genes of zebrafish remain an unsolved mystery due to the difficulty to distinguish the sex of juvenile zebrafish. However, aromatase inhibitors (AIs) could direct juvenile zebrafish sex differentiation to male and even induce ovary-to-testis reversal in adult zebrafish.

**Results:**

In order to determine the transcriptomic changes of sex differentiation in juvenile zebrafish and early sex-reversal in adult zebrafish, we sequenced the transcriptomes of juvenile and adult zebrafish treated with AI exemestane (EM) for 32 days, when juvenile zebrafish sex differentiation finished. EM treatment in females up-regulated the expression of genes involved in estrogen metabolic process, female gamete generation and oogenesis, including *gsdf*, *macf1a* and *paqr5a*, while down-regulated the expression of vitellogenin (vtg) genes, including *vtg6*, *vtg2*, *vtg4*, and *vtg7* due to the lower level of Estradiol (E2). Furthermore, EM-juveniles showed up-regulation in genes related to cell death and apoptosis, such as *bcl2l16* and *anax1c,* while the control-juveniles exhibited up-regulation of genes involved in positive regulation of reproductive process and oocyte differentiation such as *zar1* and *zpcx*. Moreover, EM-females showed higher enrichment than control females in genes involved in VEGF signaling pathway, glycosaminoglycan degradation, hedgehog signaling pathway, GnRH signaling pathway and steroid hormone biosynthesis.

**Conclusions:**

Our study shows anti-masculinization in EM-treated adult females but not in EM-treated juveniles. This may be responsible for the lower sex plasticity in adults than juveniles.

## Background

Although zebrafish is an important model species for biomedical research, its early sex differentiation genes are still unclear. Currently, the zebrafish domesticated in the laboratory is widely considered to lack sex chromosomes [1], and its sex is determined by multiple genes and largely influenced by the environment [2, 3]. As the sex of zebrafish cannot be clearly determined by morphology until 3 months post fertilization as adults, the early sex differentiation genes cannot be identified directly by comparison of genes between juvenile females and males [4].

Directed sex differentiation in the juvenile stage may provide a solution to the problem of finding early sex differentiation genes. Fish has sex plasticity and sex differentiation can be directed using sex steroids [5]. Estrogen and androgen are important to female and male differentiation, respectively. Estradiol (E2), the most effective endogenous estrogen, is biosynthesized from androgen by cytochrome P450 aromatase mainly encoded by *cyp19a1a/b* in fish [6]. Aromatase inhibitors (AIs) can thus induce male differentiation by decreasing estrogen levels [7] while increasing androgen levels in a wide variety of fish species [8, 9]. For example, transient treatment of AIs during sex differentiation causes sex reversal of the tilapia (*Oreochromis niloticus*) [10] and Japanese flounder (*Paralichthys olivaceus*) [11], manifested by the development of genotypic females into phenotypically normal males. Therefore, early sex differentiation genes can be identified through a comparison of juvenile zebrafish samples from their changes under directed sex differentiation. However, we cannot determine which individuals have undergone sex reversal.

The sex plasticity of adult zebrafish permits the possibility to distinguish early sex differentiation genes through comparison between individuals. In recent years, three independent studies have shown that adult zebrafish also retain sex plasticity. One study found that, with the disappearance of the oocyte and the retainment of glandular stem cells, the adult female zebrafish could be converted to a sperm-producing male [12]. Another study found that the transplantation of female germline cells into infertile adult males would lead to the conversion of an infertile adult male zebrafish into a fertile male [13]. The third study found that adult zebrafish underwent ovarian retraction and showed testicle-like organs after a 5-month AI treatment, followed by the production of normal sperms after another 8 weeks without AI treatment. Ovarian degeneration at 4-week treatment was the first morphological change during the sex reversal of adult zebrafish [14]. These studies indicate that the sex of adult female zebrafish can be reversed to males, while the molecular mechanisms in the reversal process have not been systematically studied.

**Fig 1:**
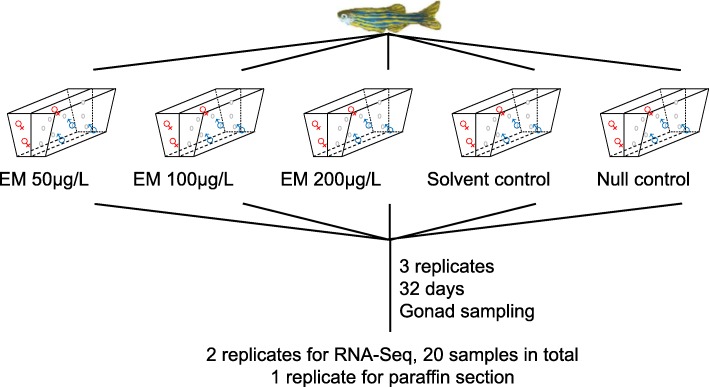
Experimental design. A general schematic overview of the zebrafish gonad RNA-Seq experiment. RNA was isolated from gonads dissected from 2 females and 2 males per group and subjected to RNA-Seq analysis

Here, in order to identify the molecular mechanisms involved in sex reversal, we performed RNA-Seq for adult zebrafish treated and untreated with AI Exemestane (EM) [15] for 32 days, when the first morphological change ovarian degeneration was induced by AI Fadrozole [14] (Fig. 1a). As a comparison, we also treated and untreated zebrafish with EM since their fertilization, and performed RNA-Seq of the juveniles at 32-day post-fertilization (dpf), when the sex differentiation was finished [16]. The genes with changed expression in EM-treated adult females and juveniles could represent the key genes in early sex differentiation and facilitate further research on sex determination in zebrafish.

## Results

### Presence of atretic follicles in adult female zebrafish after 32-day EM treatment

The effectiveness of EM was pre-tested through measuring *vtg1* expression levels of the samples after EM treatment by qRT-PCR [17]. Vtg1 was produced in the liver and was transported into the ovary for oocyte development [18], which could reflect the Estradiol (E2) level in zebrafish [19]. We detected a 2–29 fold and 343–962 fold decrease of *vtg1* expression level in the EM-females with short-term (7 days, Additional file 1) and long-term (32 days, Additional file 2) treatment, and thus confirmed the effectiveness of EM used in the study [17].

To confirm that our EM treatment can also induce male-to-female sex reversal, we exposed adult females to EM (100 μg/L) and solvent control DMSO for 90 days, according to the time for sexual maturity in larval zebrafish, and checked their change by dissection. We found that the gonads of adult female zebrafish was testis instead of ovary (Additional file 3), indicating the female-to-male sex reversal induced by our EM treatment.

**Fig 2:**
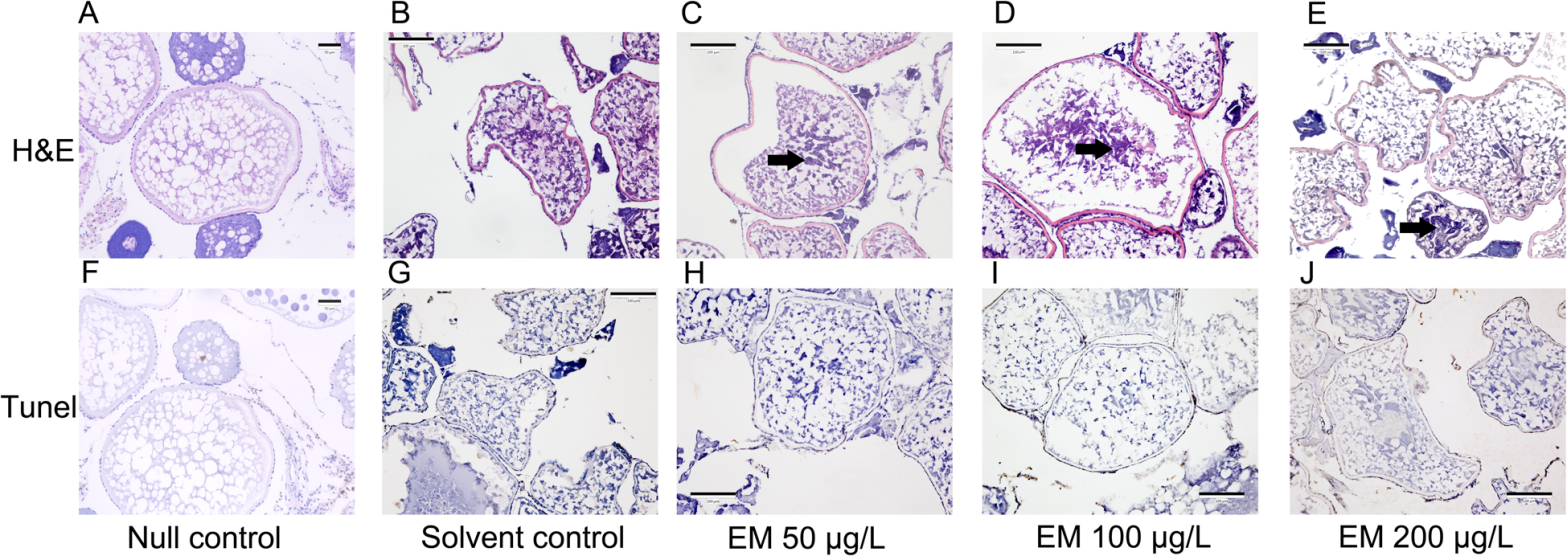
Histologic staining of gonads used in our study. **a**-**e**. Micrographs by H&E (hematoxylin-eosin) staining show ovary morphology at day 32 after treated by EM or DMSO in various treatment groups. **a**. Ovary of null control female zebrafish. **b**. Ovary of solvent control female zebrafish. **c**. Ovary of 50 μg/L EM treated female zebrafish. **d**. Ovary of 100 μg/L EM treated female zebrafish. **e**. Ovary of 200 μg/L EM treated female zebrafish; **f**-**j**. Micrographs by Tunel staining show ovary morphology at day 32 after treated by EM or DMSO in various treatment groups. **f**. Ovary of null control female zebrafish. **g**. Ovary of solvent control female zebrafish. **h**. Ovary of 50 μg/L EM treated female zebrafish. **i**. Ovary of 100 μg/L EM treated female zebrafish. **j**. Ovary of 200 μg/L EM treated female zebrafish. The arrow pointing is the position of the chromatin condensation in follicles of EM-females in C-E

We then exposed female and male adult zebrafish to low concentration (50 μg/L), intermediate concentration (100 μg/L), high concentration (200 μg/L) EM, solvent control DMSO and null control without EM or DMSO for 32 days to induce female-to-male sex reversal (Fig. 1). Neither decreased body weight nor width-to-length ratio of EM-females was observed (Additional file 4). Also, no obvious changes in gonads were found by dissection between females treated and untreated with EM for 32 days. We further observed the gonads in paraffin section, and found the deep staining caused by chromatin condensation in follicles of EM-females (Fig. 2a-e), demonstrating atretic follicles. Although atretic follicles might be related to apoptosis, TUNEL staining showed no evidence of apoptosis in EM-females (Fig. 2f-j). These observations above were inconsistent with the ovarian degeneration of adult females after four-week treatment of another AI Fadrozole [14], implying the differences in drug efficiency (EM vs Fadrozole) and/or treatment approaches (soaking vs feeding).

### Small transcriptional perturbations caused by 32-day EM treatment

**Fig. 3:**
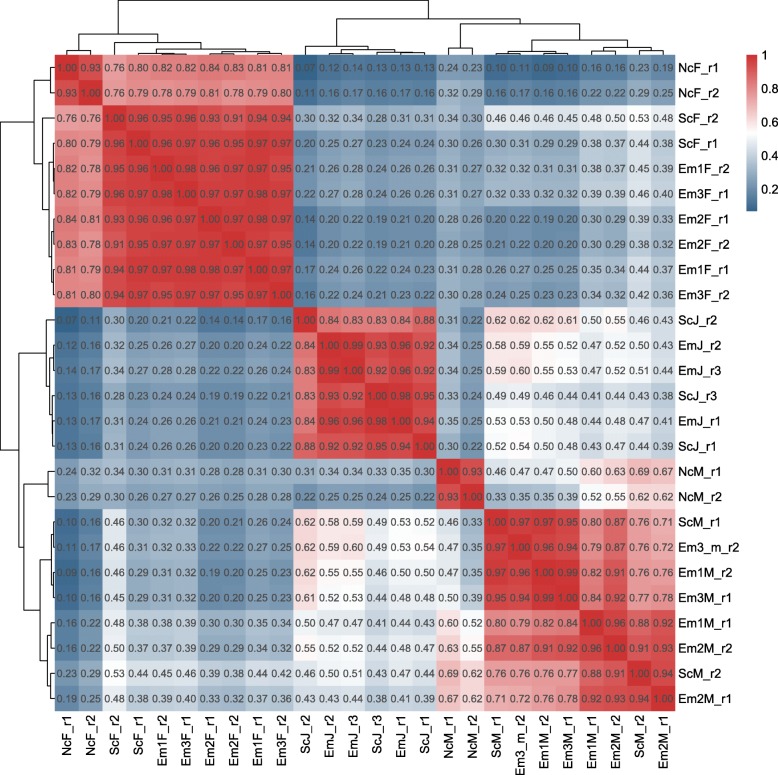
The correlation and hierarchical clustering of all the samples used in our study. Expression level was measured by read counts which were normalized by DESeq2. ScF: Gene expression level of ovary of solvent control female zebrafish; ScM: Gene expression level of testis of solvent control male zebrafish; ScJ: Gene expression level of solvent control juvenile zebrafish; EM1F: Gene expression level of ovary of of 50 μg/L EM (exemestane) treated female zebrafish; EM2F: Gene expression level of ovary of of 100 μg/L EM treated female zebrafish; EM3F: Gene expression level of ovary of of 200 μg/L EM treated female zebrafish; EM1M: Gene expression level of testis of of 50 μg/L EM treated male zebrafish; EM2M: Gene expression level of testis of of 100 μg/L EM treated male zebrafish; EM3M: Gene expression level of testis of 200 μg/L EM treated male zebrafish; EM-j: Gene expression level of juvenile zebrafish treated by 10 μg/L EM; NcF: Gene expression level of ovary of fish-water without adding neither DMSO or EM female zebrafish; NcM: Gene expression level of ovary of fish-water without adding neither DMSO or EM male zebrafish. r: replicate

To identify the molecular mechanisms underlying the early transition from female to male, we dissected the gonads from two females and two males exposed to the low, intermediate, high concentrations of EM, solvent control or null control, and performed RNA-Seq. As a comparison with early transition of juveniles, we also included pools of whole body juvenile samples treated with and without EM for 32 days after fertilization. A total of 20 adult samples and 6 juvenile samples were sequenced with the mapping efficiency ranging from 78.21 to 92.67% (Additional file 5). The correlation coefficients between replicates were 0.76–0.99, showing satisfactory repeatability (Fig. 3).

Hierarchical clustering showed that three major groups corresponded to adult females, adult males, and juveniles, respectively. The juvenile group clustered closer to the adult male group rather than the adult female group. In each group, samples treated with EM were clustered together with solvent control instead of null control, demonstrating small perturbations caused by DMSO (Fig. 3). The high correlation of gene expression between EM treated zebrafish and their solvent control in each group demonstrating little side effects induced by the 32-day EM treatment, allowing us to further detect the early transcriptional changes in the sex reversal. An extended treatment time might produce more detectable expression differences.

### Anti-masculinization induced by EM treatment in adult females

To identify the key genes responsible for the early sex transition from female to male in adults, we first explored the differentially expressed genes (DEGs) between solvent control females and solvent control males (DEG_scF-scM_) as a reference. We found a total of 3927 solvent-control-female-biased genes and 6797 solvent-control-male-biased genes (Additional file 6A, Additional files 7, 13). The larger number of solvent-control-male-biased genes than solvent-control-female-biased genes was consistent with the sex-biased gene expression patterns among most species. We validated the sex bias of some known female-biased genes, including *vtg1* and *gdf9* [17, 20] and male-biased genes, including glyceraldehyde-3-phosphate dehydrogenase spermatogenic (*gapdhs*) [21] and anti-mullerian hormone (*amh*) [20]. As expected, Gene Ontology (GO) analysis also revealed that female-biased genes were enriched in female-specific functions like binding of sperm to zona pellucida, sperm-egg recognition, egg coat formation, and regulation of acrosome reaction, while male-biased genes were enriched in male-specific functions like cilium organization, movement and assembly (Additional file 6 BC).

As another reference, we explored the DEGs between EM-males and solvent control males (DEG_scM-emM_). A total of 141 DEG_scM-emM_ were found, including 100 up-regulated genes and 41 down-regulated genes in EM-male (Additional file 6 D, Additional files 8, 13). The GO analysis showed the up-regulated genes in EM-males were enriched in the GO terms related to ruffle assembly and organization (Additional file 6E), which were reported to be involved in fatty acid metabolism [22]. This suggested the abnormality of metabolic pathways in EM-males relative to control males, probably due to the changes in their metabolic functions to accelerate drug metabolism in response to the drug treatment.

**Fig. 4:**
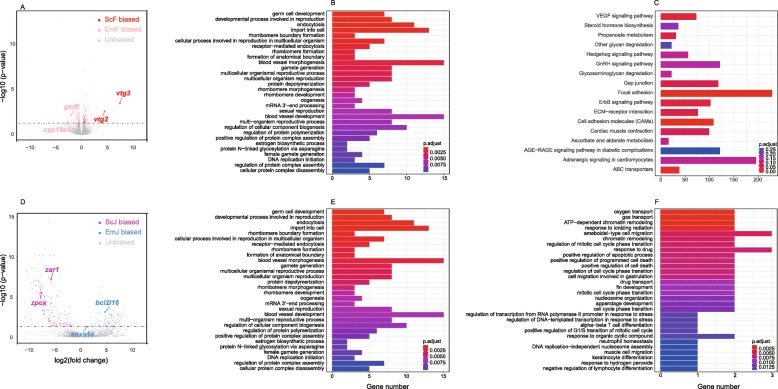
The gene expression profile in EM treated and solvent control adult female zebrafish group and juvenile zebrafish group. **a**. Comparison of differentially expressed gene in control female and EM-treated female gonad. Volcano plot shows genes control female ovary biased expression and EM-treated female ovary biased expression. **b**. EM-treated female-biased gene GO (Gene Ontology) enrichment analysis. The length of the bar corresponds to the number of genes enriched in the corresponding GO terms, and the color from red to blue represents the p.ajust value changes. **c**. GSEA (Gene set enrichment analysis) based on genes expressed in EM-treated female. The length of the bar corresponds to the number of genes enriched in the corresponding GO terms, and the color from dark blue to light blue represents the p.ajust value changes. **d**. Comparison of differentially expressed gene in control juvenile and EM-treated juvenile. Volcano plot shows genes control juvenile biased expression and EM-treated juvenile biased expression. **e**. GO enrichment analysis based on genes which show control juvenile biased expression. **f**. GO enrichment analysis based on genes which show EM-treated juvenile biased expression

We next explored the DEGs between EM-females and solvent control females (DEG_emF-scF_). The effects of EM concentration were tested through comparisons among EM-females treated with low, intermediate, and high concentrations of EM. Only 134, 122 and 131 DEGs were found in the low-intermediate, low-high, and intermediate-high concentration comparisons. We thus compared all the six EM-females of different concentrations together with solvent control females, and found 548 DEG_emF-scF_, including 370 up-regulated genes and 178 down-regulated genes in EM-females (Fig. 4a, Additional files 9, 13). As a reference, we also explored the DEGs between solvent control females and null control females (DEG_scF-ncF_) and found 7512 DEG_scF-ncF_, including 3575 up-regulated genes and 3937 down-regulated genes in solvent control females (Additional files 10, 13). Vtg genes, including *vtg6*, *vtg2*, *vtg4*, and *vtg7,* were down-regulated in EM-females relative to both solvent control females and null control females, supporting lower endogenous E2 level caused by EM treatment.

The function of DEG_emF-scF_ was further investigated through GO analysis and GSEA. Surprisingly, the DEG_emF-scF_ up-regulated in EM-females were enriched in the GO terms mainly related to ovarian functions, like estrogen metabolic process, female gamete generation and oogenesis (Fig. 4b). Meanwhile, the DEG_scM-emM_ up-regulated in EM-males were enriched in none of these terms, suggesting at least partial anti-masculinization induced by EM treatment in females to maintain the endogenous E2 level through activation of the hypothalamic-pituitary-gonad [23]. The GSEA result showed that genes upregulated in EM-females were enriched in 17 KEGG pathways, which are mainly involved in signal transduction and metabolic pathways (Fig. 4c, Additional file 11). Most of the up-regulated pathways have been reported to be driven by gonadal steroid, especially for the androgen in Atlantic cod (*Gadus morhua L.*) [24].

**Fig. 5:**
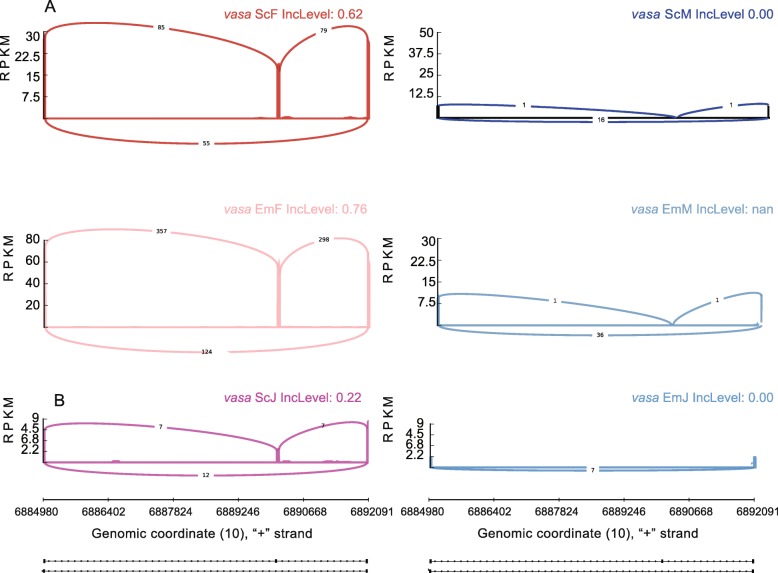
The different isoforms of gene *vasa* in samples we used. **a**. The different isoforms of gene *vasa* in adult EM-treated and solvent control zebrafish. **b**. The different isoforms of gene *vasa* in juvenile EM-treated and solvent control zebrafish

To confirm the anti-masculinization in EM-females, we examined the alternative splicing of vasa, a molecular sex marker with sex-specific isoforms. The *vasa* is a conserved gene involved in spermatogenesis and germ cell formation in mice and flies, and is essential for sex differentiation and gamete formation in zebrafish. It has two isoforms in females, while only the shorter isoform lacks exon 4 in males. The isoform of *vasa* with the exon 4 was transcribed in future female zebrafish at 20–25 dpf, leading to apoptosis in future male zebrafish [16, 25]. Alternative splicing of vasa using rmats [26] showed an increased ratio of longer isoform in EM-females (Fig. 5a), supporting the anti-masculinization in EM-females.

### EM induces faster sex differentiation to male in juveniles

To compare the transcriptomic changes between juveniles and adult females after 32-day EM treatment, we explored the DEGs between EM-juveniles and control juveniles (DEG_scJ-emJ_) and found 263 DEG_scJ-emJ_ (Fig. 4d, Additional files 12, 13). DEG_scJ-emJ_ up-regulated in control juveniles were enriched in the biological processes related to positive regulation of reproductive process, sperm-egg recognition, and oocyte differentiation (Fig. 4e). These observations suggested that EM induced the sex differentiation of juveniles to male. Furthermore, GO analysis showed that the DEG_scJ-emJ_ up-regulated in EM-juveniles were enriched in the biological processes related to cell death and apoptosis [27], implying that the progress of EM-induced sex differentiation to male was faster in juveniles than in adult females (Fig. 4f).

We further checked the alternative splicing of vasa between EM-juveniles and control juveniles, and found, in contrast to adult EM-females with a decreased ratio of shorter isoform, EM-juveniles only expressed the shorter isoform of vasa, demonstrating the lack of feminization in EM-juveniles with the same treatment duration (Fig. 5b).

## Discussion

We explored the molecular mechanisms underlying the early sex differentiation in zebrafish by exploring transcriptomic changes in the early stage of sex reversal in adults and juveniles after 32-day EM treatment. In this discussion, we focused on the rationality of our strategy. We also discussed the differences in sex plasticity between adults and juveniles as notes for reference in using sex reversal models.

### Rationality of using adult sex reversal model to study early sex differentiation

Fish sex is highly plastic. The germline stem cells exist both in juveniles and adults [12–14]. The down-regulation of endogenous estrogen induces female trans-differentiation in juvenile and adult fish [17, 28]. Impairing androgen synthesis and elevating exogenous estrogen can induce the male trans-differentiation in adult tilapia while exogenous estrogen alone can direct sex differentiation to female in juvenile fish [29]. These observations indicate the effects of sex hormone in the sex differentiation and maintenance in fish [30].

The germline stem cells in juvenile adult female zebrafish could differentiate into male after AI treatment by regulating sex hormone levels [14], we could thus identify DEGs between juveniles or adult females treated and untreated with AI EM to explore the mechanisms underlying early sex differentiation. Comparing with juvenile model, the adult sex reversal model has unique advantages in the feasibility of sex identification and gonad manipulation.

### Lower sex plasticity in adults than juveniles

Sex differentiation of juvenile zebrafish, both future females and future males, is first involved in the development of an ovary (called “juvenile ovary”) [31]. Oocyte loss and ovary lumen breakdown by apoptosis at 21–25 dpf are male-specific events with testicular differentiation [31]. In females, early diplotene oocytes apoptosis was found in the juvenile ovary at 15–19 dpf, indicating a programmed loss of oocytes during ovarian development to eliminate unnecessary oocytes from the ovary [16]. In male zebrafish, all oocytes disappeared from gonads within 30 dpf and spermatocytes developed with the differentiation of testis, while female oocytes in the ovaries become mature [16, 31].

Sex hormone treatment can induce directed sex differentiation and sexual reversal of the differentiated genital organs in zebrafish [14]. In the AI-induced directed sex differentiation in juvenile zebrafish, apoptotic oocytes of early diplotene and perinucleolar stages could be observed even at 15–40 dpf [27], longer than that in untreated juveniles. Besides, individuals display phenotypically male gonads at 71 dpf [28]. However, in the AI-induced female-to-male sex reversal, the ovary structure develops into testis and produces mature sperm after 5 months treatment in adult females [14], indicating poorer sex plasticity in adults than in juveniles.

In this study, we found the up-regulation of genes related to apoptosis in juveniles after 32-day EM treatment, consistent with the effects of EM on sex differentiation [27], suggesting that EM may postpone ovarian apoptosis in juvenile zebrafish. Meanwhile, no evidence of ovarian apoptosis were observed between EM-females and control females over 32 days post spawning, suggesting slower sex differentiation and lower sex plasticity in adults than juveniles with EM treatment. Furthermore, the anti-masculinization in transcriptomes we observed in EM-females, relative to control females, supporting again the lower plasticity in adults than juveniles and providing the underlying mechanisms.

## Conclusion

Adult female zebrafish exhibit lower sex plasticity than juveniles due to anti-masculinization in adult zebrafish. This study provides new insights into the sex differentiation in zebrafish.

## Methods

### Ethics approval and consent to participate

The zebrafish used in this study were purchased from China Zebrafish Resource Center. All animal husbandry and procedures were approved by National Research Council (NRC) and performed strictly according to the guidelines set for the usage of animals by this committee.

### Zebrafish husbandry

Zebrafish (AB strain) were obtained from China Zebrafish Resource Center and maintained at 28.5 °C with a light/dark cycle of 14/10 h.

For each juvenile sample, fifty embryos of zebrafish were maintained in a petri dish from 0 dpf, and transferred to a three-litter tank in the recirculation systems at 5 dpf. They were fed with paramecium at 5–15 dpf and fairy shrimp at 16–32 dpf twice a day.

For adult samples, three pairs of adult zebrafish were kept in a three-liter tank in the recirculation systems and fed twice a day with fish food (Zeigler AP100). Male and female adult zebrafish were distinguished according to their morphology, i.e., the females are fatter with a lighter color while the males are slender with a yellowish color. The sex of the adult zebrafish was further confirmed through their mating behavior at 90 dpf. The two sexes of adult zebrafish were separated on a transparent acrylic plate with evenly distributed holes to ensure the same treatment for all the fish samples in the same tank.

In this study, 30 adult zebrafish and 150 juvenile zebrafish were used, including 10 adult zebrafish for histological examination, 20 adult zebrafish for transcriptome sequencing, and 150 juvenile zebrafish were divided into 6 samples for transcriptome sequencing.

### Exemestane treatment

The AI EM (6-methylenandrosta-1, 4-diene-3, 17-dione, ≥ 98% purity, Bervita) used for adult and juvenile zebrafish was dissolved in 100 μg/L and 10 μg/L DMSO, respectively. Five groups of adult zebrafish were raised in continuous exposure to different concentrations of EM (50 μg/L, 100 μg/L and 200 μg/L), the solvent control (DMSO) or null control. Meanwhile, two groups of juvenile zebrafish were also raised in continuous exposure to 10 μg/L EM or the solvent control. We chose the EM concentration 10 μg/L for larval zebrafish because such concentration of another AI Fadrozole could direct sex differentiation to male and higher concentration of Fadrozole would reduce the survival rate of larval zebrafish [32] . Each adult group included a tank of zebrafish (three pairs of adult zebrafish) while each juvenile group included three tanks of zebrafish (each tank was raised from 50 embryos in a petri dish). All the groups were exposed for 32 days. The water in the tanks was refreshed every day with an equivalent concentration of EM or DMSO to ensure the effectiveness of drugs. The fish were anesthetized by immersion in 40 mg/L of tricaine methane sulfonate (MS-222, Sigma-Aldrich) to collect the gonad and were euthanized by immersion into a 0.5 g/L tricaine solution at the end of the experiment.

### Validation of estrogenic marker vitellogenin 1 (*vtg1*) in EM-females

We tested the effectiveness of EM with *vtg1* expression after short-term EM treatment. In the short-term treatment, EM was dissolved in 100 μg/L DMSO. Four groups of adult female zebrafish were exposed for 7 days to EM with the concentration 0 μg/L, 50 μg/L, 200 μg/L and 800 μg/L, separately. Each group contained three replicates, with one zebrafish per replicate. The water was refreshed every day with an equivalent concentration of EM to ensure the effectiveness of EM.

Quantitative real-time PCR (qRT-PCR) of *vtg1* was conducted on the whole body of 12 adult female zebrafish treated with the four concentrations of EM for 7 days. Total RNA were extracted from the somatic tissues by Trizol (Invitrogen) and incubated with RNase-free DNase I (Takara) at 37 °C to remove the genomic DNA. The reaction was terminated by adding 50 mM EDTA at 65 °C for 10 min. After determining the concentration of RNA by spectrophotometry (Thermo), 1000 ng of total RNA was reverse transcribed into cDNA with Revert Aid First Strand cDNA Synthesis Kit (Thermo). The resulting cDNA was used as the template in the PCR system, and *vtg1* expression was tested using the primers vtg1-F (5′-ACGAACAGCGAGAAAGAGATTG-3′) and vtg1-R (5′-GATGGGAACAGCGACAGGA-3′) [33]. *18 s* was amplified as an internal control using forward (5′- CGGAGGTTCGAAGACGATCA-3′) and reverse primers (5′- TCGCTAGTTGGCATCGTTTATG-3′) [12]. The relative gene expression levels were determined based on the reference gene *18 s* using the comparative method (ΔΔCt).

The expression levels of *vtg1* in somatic tissues of 16 samples treated with different concentrations of EM for 32 days were also checked by qRT-PCR with the same protocol. However, the internal control gene was changed to *β-actin* using the forward primer (5′-ATGGATGAGGAAATCGCTGCC) and reverse primer (3′-CTCCCTGATGTCTGGGTCGTC) [33].

### Histology and TUNEL staining

The zebrafish gonads of one female and one male per adult group were dissected for histological examination. All the female samples were fixed by immersing the samples in 10% buffered formalin in PBS for 24 h. The tissues were paraffin-embedded, processed for light microscopy and sectioned at 4 μm in thickness. The sections were stained with hematoxylin/eosin for histopathological examination. The apoptosis in the ovary was examined by the TUNEL assay using TdT in situ apoptosis detection DeadEnd™ Colorimetric TUNEL System (G7360) from Promega according to the instructions provided by the manufacturer [34].

### RNA extraction and sequencing

The zebrafish gonads of two females and two males per adult group were dissected for RNA-Seq of the adults (Fig. 1). RNA samples from each individual were exacted using Trizol (Invitrogen) according to the manufacturer’s protocol. Stranded PolyA+ RNA libraries were prepared at BGI with in-house kits. The library quality was assessed by checking the distribution of the fragment size using Agilent 2100 bioanalyzer (Agilent) and the quantity of the libraries was measured using qRT-PCR (TaqMan Probe). A total of 16 qualified strand-specific cDNA libraries were constructed and were sequenced on the Illumina HiSeq XTen System (Illumina). High-throughput sequencing raw image data files were converted to reads using CASAVA Base Calling (bcf2fastq) analysis.

The whole bodies of 25 zebrafish from a tank per sample and three samples per treatment were used for RNA-Seq of the juveniles. All the six RNA samples were extracted and sequenced as adult zebrafish except that the sequencing libraries were prepared at Annoroud using VAHTS Stranded mRNA-seq Library Prep Kit (Vazyme Biotech) following the official protocol.

### Read mapping and quantification

The quality of all the raw reads was evaluated using FastqC (0.11.8) and sequencing report tool of MultiQC (0.9) [35]. Reads were mapped to the corresponding reference genome (Ensembl v95) using Hisat2 (2.0.5) with parameters “--dta -x --rna-strandness RF” and “--known-splicesite-infile”, followed by gene annotation in GTF format (Ensembl v95) [36]. The Hisat2 data were converted to BAM format and sorted by Samtools (1.5), and PCR duplicates were removed using Samtools command rmdup –S [37]. HTSeq (0.9.1) was used for counting reads with parameters “-t exon –i gene_id -r pos -s reverse” [38].

### Identification and enrichment analysis of differentially expressed genes (DEGs)

DEGs in the zebrafish gonads were identified across eight different groups (two sexes and four treatments per sex for adults) using DESeq2 (1.22.1) [39]. Genes with adjusted *p*-value < 0.05 were considered as differentially expressed [40]. The same protocol was used for juvenile zebrafish to identify DEGs between two groups with and without EM treatment [41].

Biological functions and metabolic processes among all the DEGs were identified using the tools org. Dr.eg.db (3.7.0) [42], clusterprofiler (3.10.1) [43] and Gene set enrichment analysis (GSEA) (3.0) [44, 45].

The differentially alternative spliced genes in zebrafish gonads were identified and visualized by rMATs (4.0.2) with paramenters “-t paired –readLength 150 –libType fr-firststrand” [26] and rmats2sashimiplot [46], respectively.

## Supplementary information

**Additional file 1. ***Vtg1* expression level significantly decreased in the EM-females treated by EM for short-term 7 days.

**Additional file 2. ***Vtg1* expression level significantly decreased in the EM-females treated by EM for long-term 32 days.

**Additional file 3.** The morphology, body weight and length of EM treated female and zebrafish in solvent control group. A. Morphology of EM treated female and zebrafish in solvent control group. B. Body weight distribution of zebrafish in treatment group and solvent control group. C. Body length distribution of zebrafish in treatment group and solvent control group.

**Additional file 4.** Histologic staining of gonads treated by 100 μg/L EM or solvent for 90 days. A. Histologic staining of adult female zebrafish gonads treated by 100 μg/L EM for 90 days. B. Histologic staining of of adult female zebrafish gonads treated by solvent for 90 days.

**Additional file 5.** The mapping efficiency rate of 24 adult samples and 6 juvenile samples sequencing reads.

**Additional file 6.** The gene expression profile in solvent control adult zebrafish group and EM-treated and solvent control adult male group. A. Comparison of differentially expressed gene in control male and female gonad. Volcano plot shows genes control female ovaries biased expression and control male testis biased expression. B-C. GO enrichment analysis based on genes which show sex-biased expression in control females and males. B. Female-biased gene GO enrichment analysis. C. Male-biased gene GO enrichment analysis. D. Comparison of differentially expressed gene in control male and EM-treated male gonad. Volcano plot shows genes control male testis biased expression and EM-treated male testis biased expression. E. GO enrichment analysis based on genes which show EM-treated male testis biased expression. The length of the bar corresponds to the number of genes enriched in the corresponding GO terms, and the color from red to blue represents the p.ajust value changes.

**Additional file 7.** The list of differentially expressed genes (DEGs) between solvent control females and solvent control males

**Additional file 8.** The list of differentially expressed genes (DEGs) between solvent control males and EM treated male.

**Additional file 9.** The list of differentially expressed genes (DEGs) between solvent control females and EM treated females.

**Additional file 10.** The list of differentially expressed genes (DEGs) between solvent control females and null control females

**Additional file 11.** The GSEA result of genes upregulated in EM-females compared to control females.

**Additional file 12.** The list of differentially expressed genes (DEGs) between solvent control juveniles and EM treated juveniles.

**Additional file 13.** The summary of differently expressed genes in all the groups.

## Data Availability

Data from all 20 adult samples and 6 juvenile samples are available at the Gene Expression Omnibus (GEO) under accession (GSE142355).

## Abbreviations

DEGs: Differentially expressed genes
DEG_scF-ncF_: Differentially expressed genes between solvent control females and null control females
DEG_scF-scM_: Differentially expressed genes between solvent control females and solvent control males
DEG_scM-emM_: Differentially expressed genes between EM-males and solvent control males
EM: Exemestane
H&E: hematoxylin-eosin
DEG_emF-scF_: Differentially expression genes between EM-females and solvent control females.
DEG_scJ-emJ_: Differentially expression genes between EM-juveniles and solvent control juveniles
GSEA: Gene set enrichment analysis
GO: Gene ontology
