# Supplementary figures and images for: Anti-masculinization induced by aromatase inhibitors in adult female zebrafish
Source: BMC Genomics. 2020 Jan 7;21:22. doi: 10.1186/s12864-019-6437-z (PMC6947999; doi:10.1186/s12864-019-6437-z)

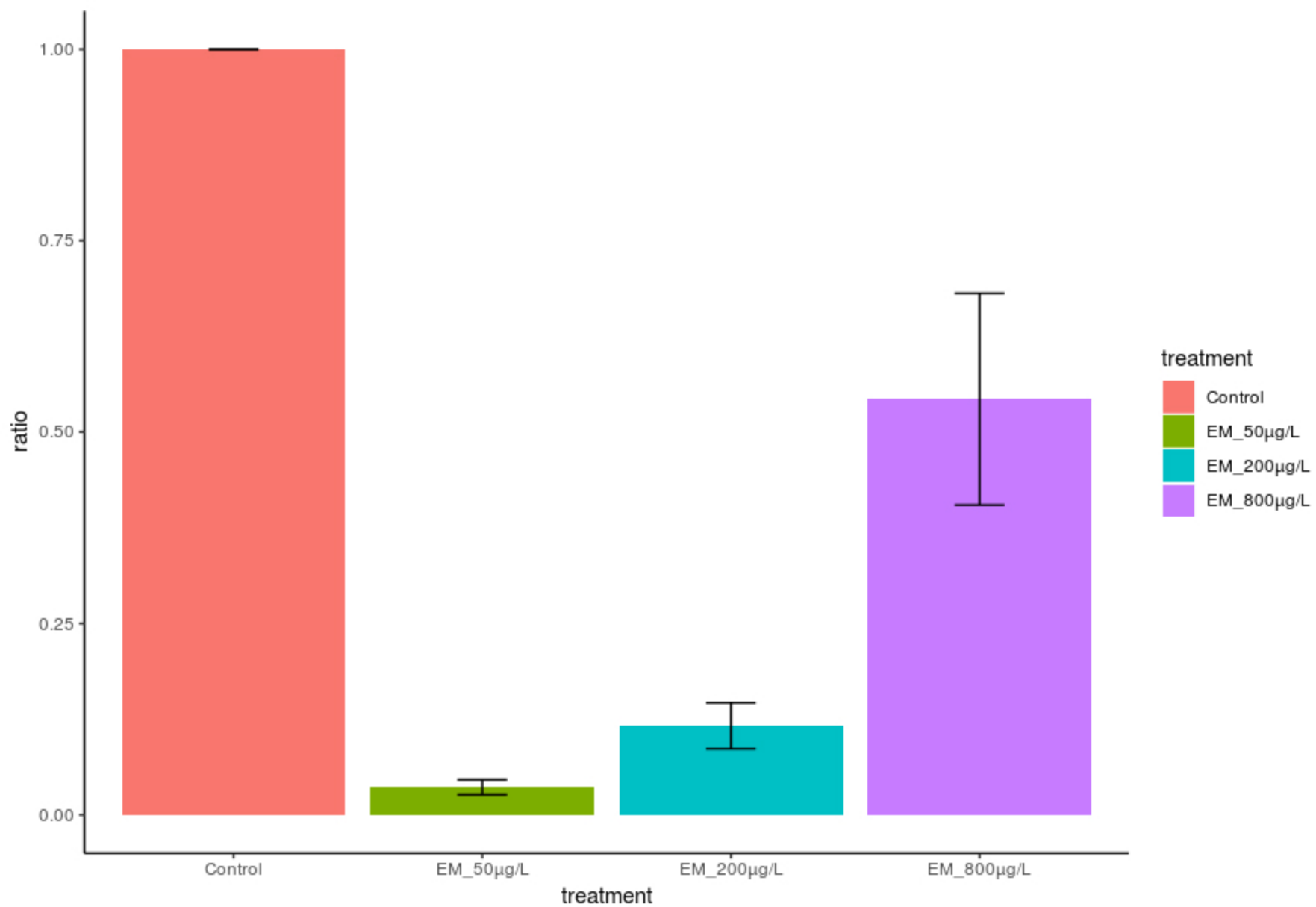

Supplement: Supplementary file 1 — Additional file 1. Vtg1 expression level significantly decreased in the EM-females treated by EM for short-term 7 days. [file 12864_2019_6437_MOESM1_ESM.pdf]

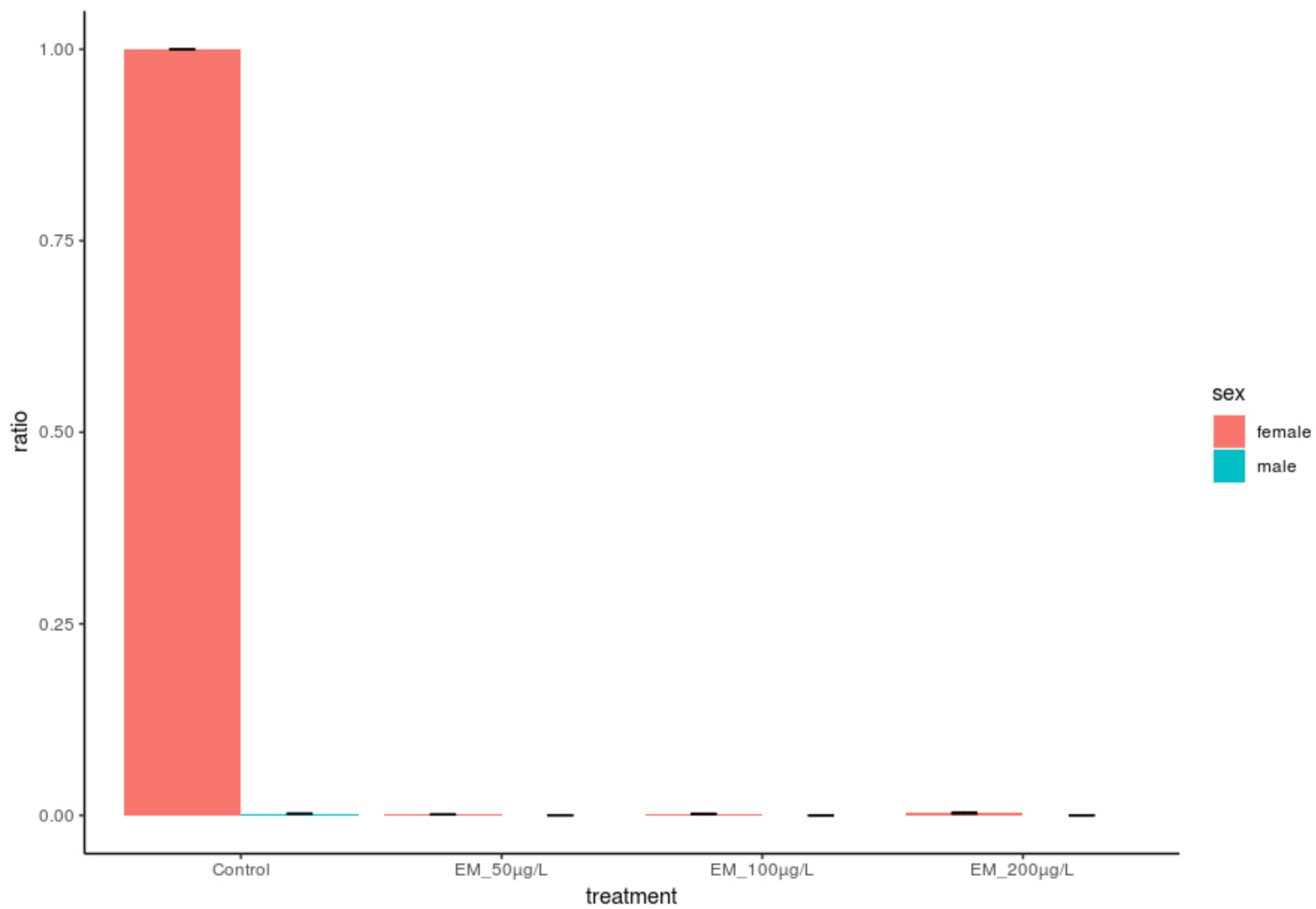

Supplement: Supplementary file 2 — Additional file 2. Vtg1 expression level significantly decreased in the EM-females treated by EM for long-term 32 days. [file 12864_2019_6437_MOESM2_ESM.pdf]

A

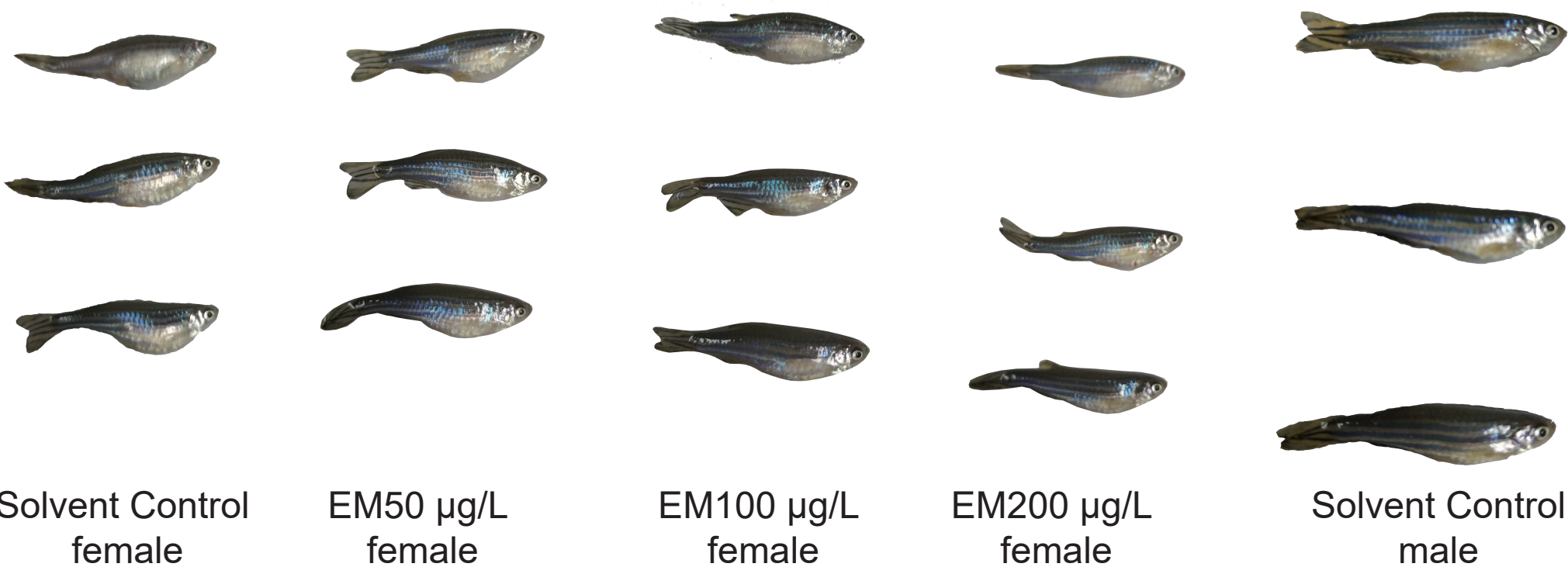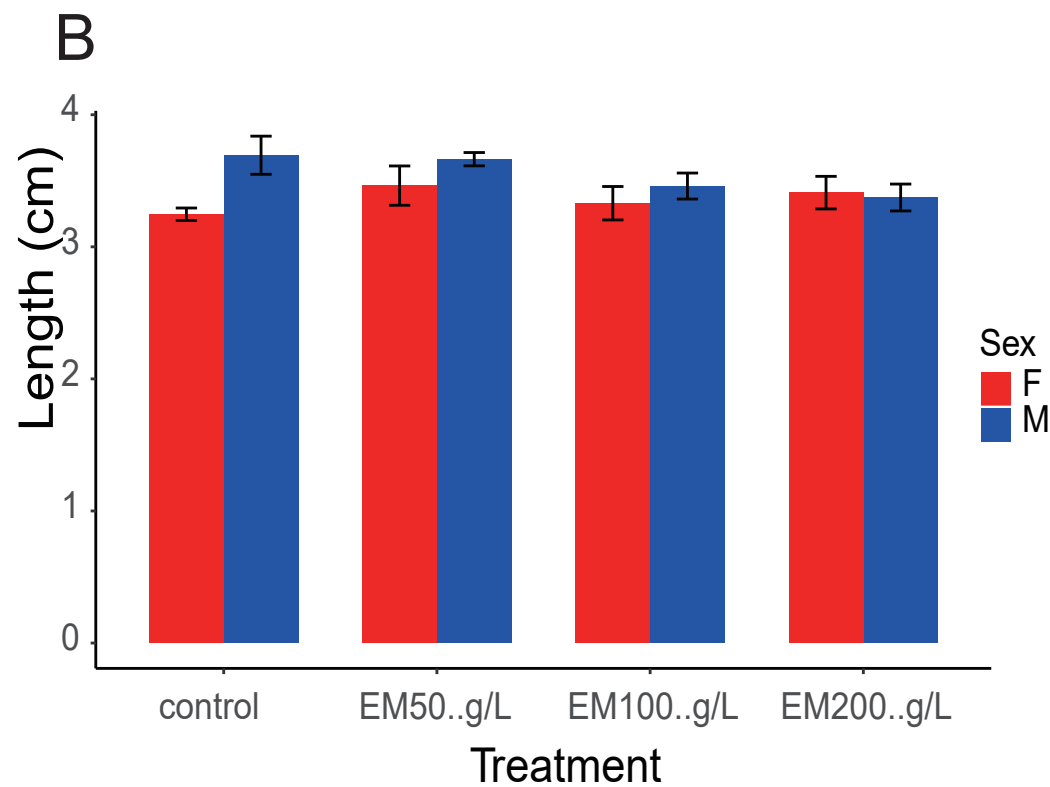

C

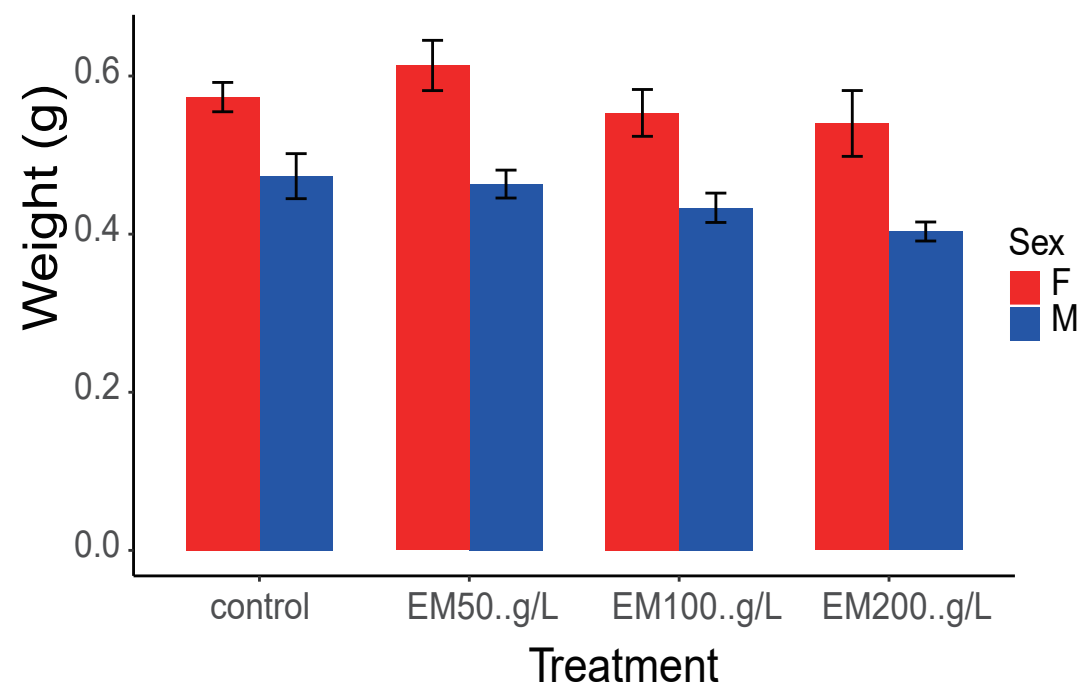

Supplement: Supplementary file 3 — Additional file 3. The morphology, body weight and length of EM treated female and zebrafish in solvent control group. A. Morphology of EM treated female and zebrafish in solvent control group. B. Body weight distribution of zebrafish in treatment group and solvent control group. C. Body length distribution of zebrafish in treatment group and solvent control group. [file 12864_2019_6437_MOESM3_ESM.pdf]

**A**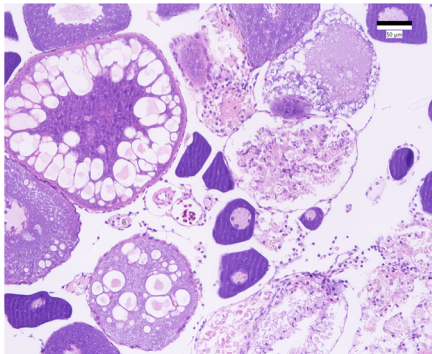**B**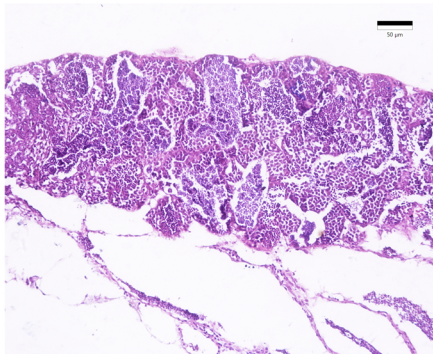

Supplement: Supplementary file 4 — Additional file 4. Histologic staining of gonads treated by 100 μg/L EM or solvent for 90 days. A. Histologic staining of adult female zebrafish gonads treated by 100 μg/L EM for 90 days. B. Histologic staining of of adult female zebrafish gonads treated by solvent for 90 days. [file 12864_2019_6437_MOESM4_ESM.pdf]

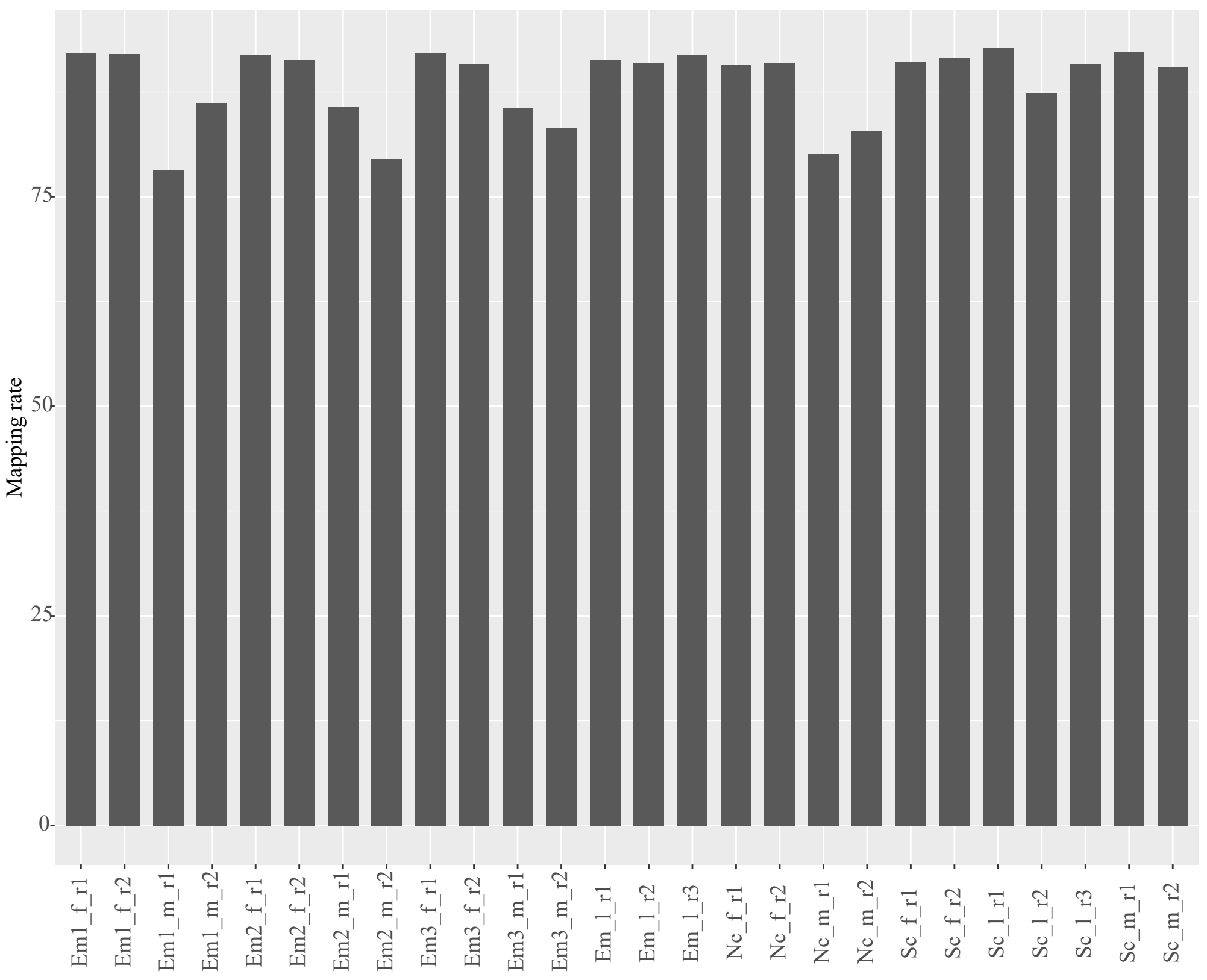

Supplement: Supplementary file 5 — Additional file 5. The mapping efficiency rate of 24 adult samples and 6 juvenile samples sequencing reads. [file 12864_2019_6437_MOESM5_ESM.pdf]

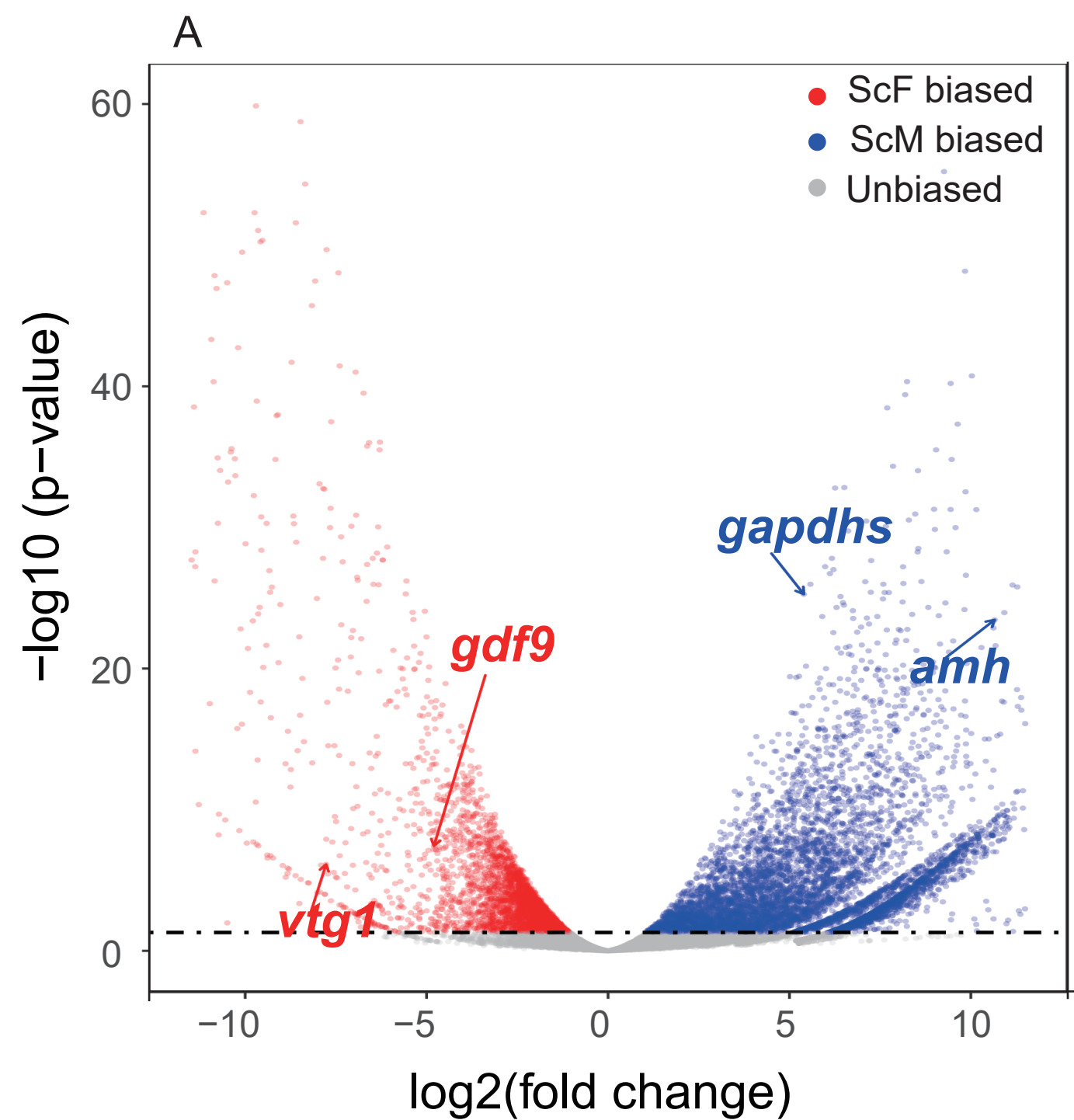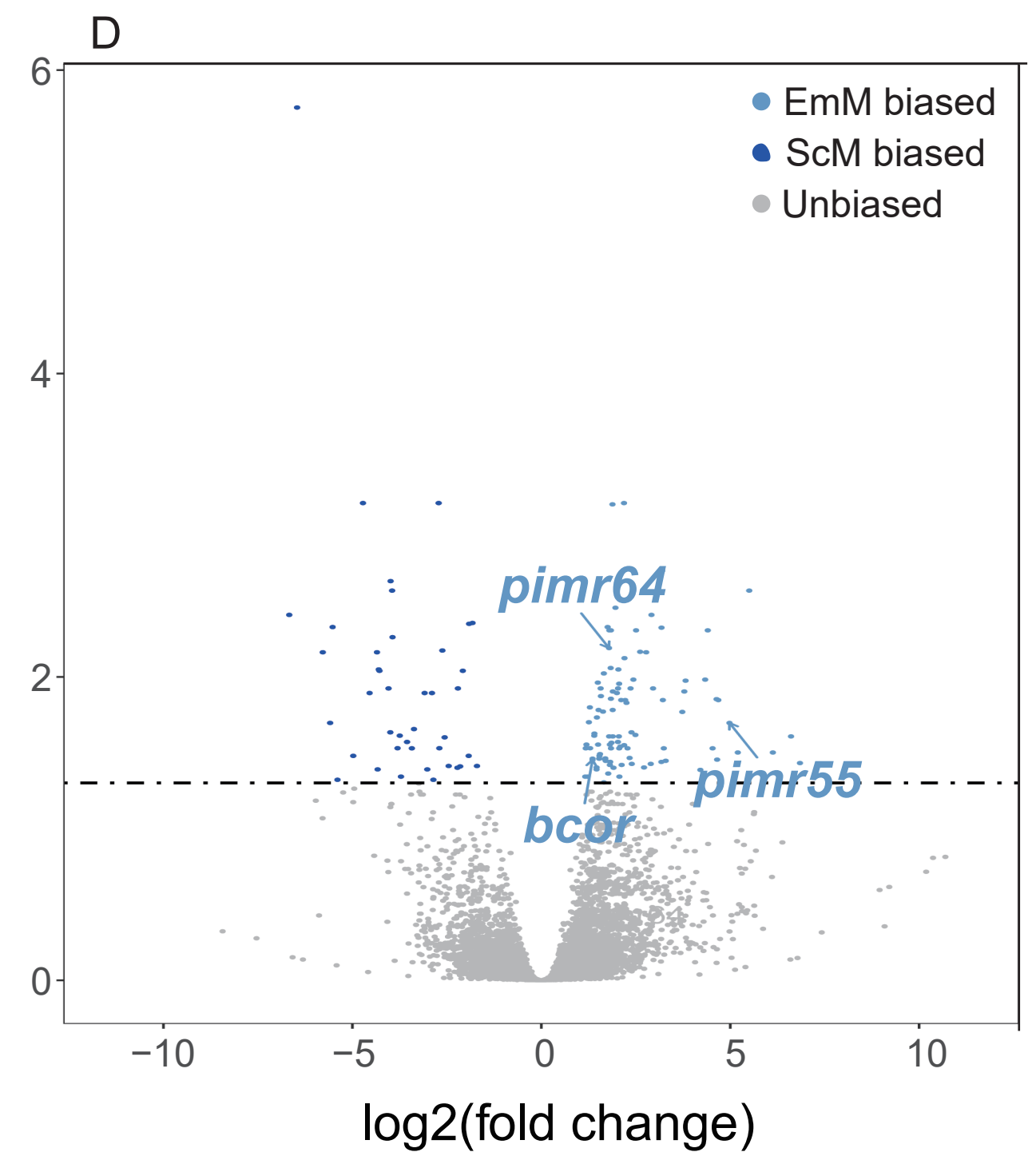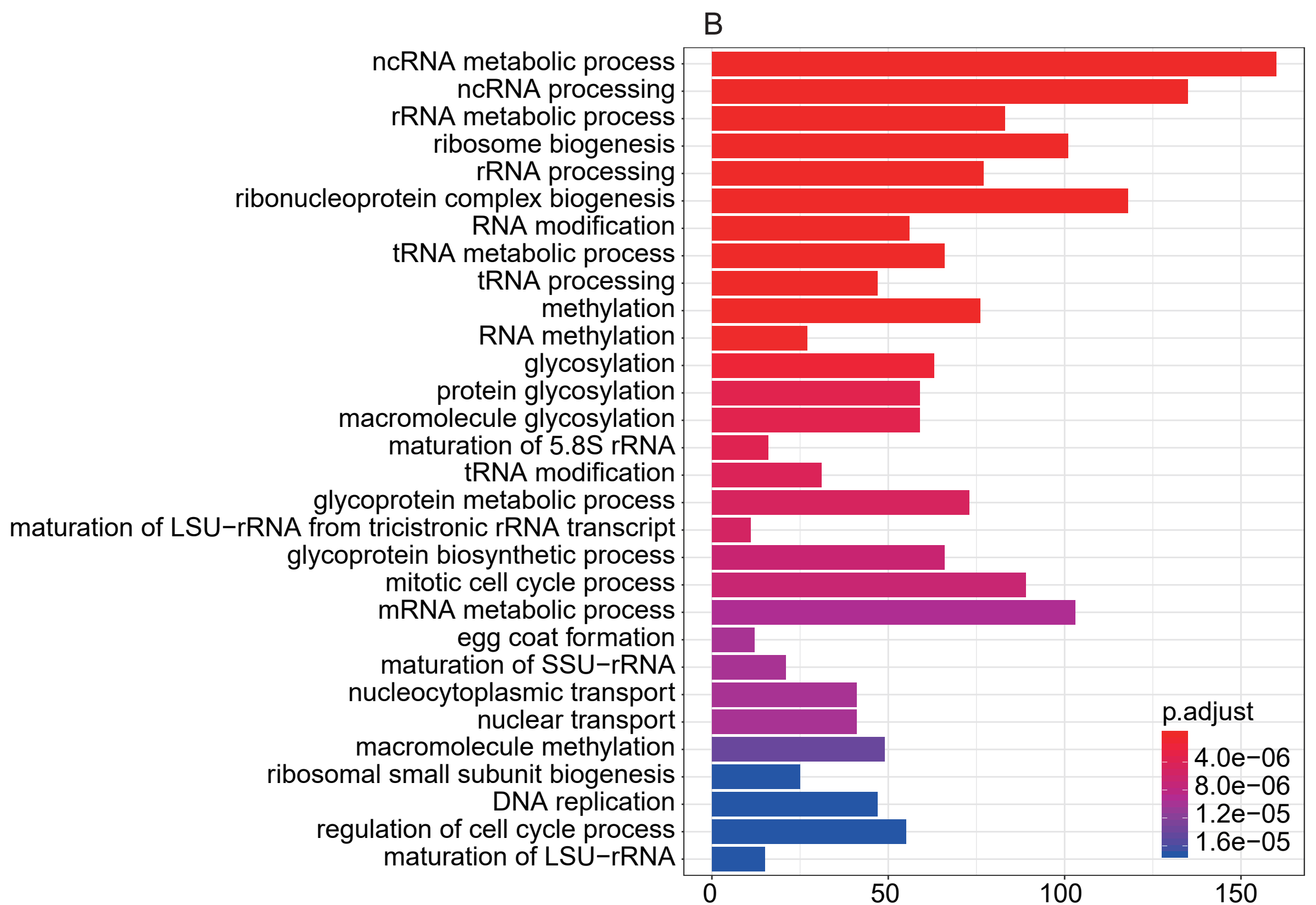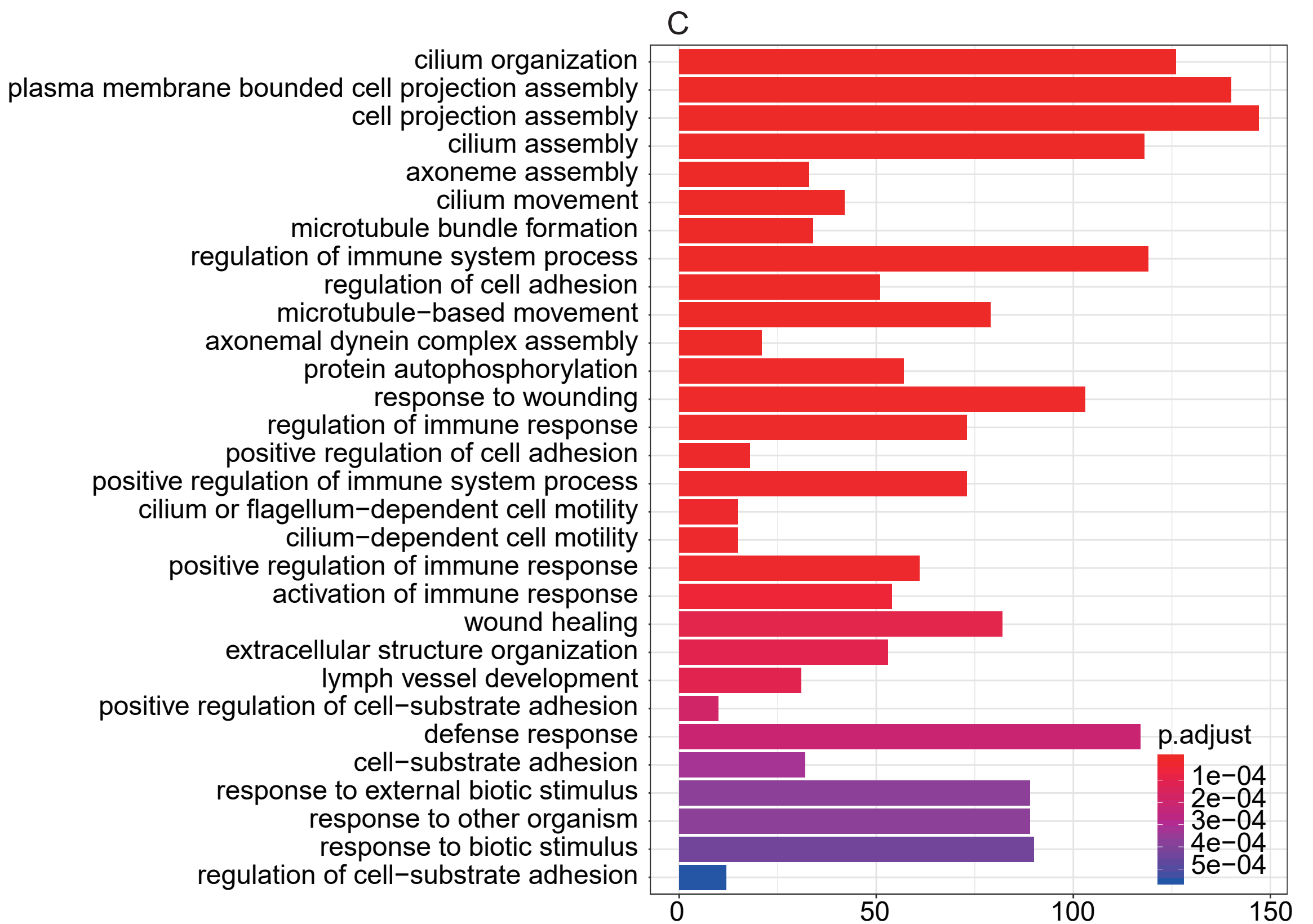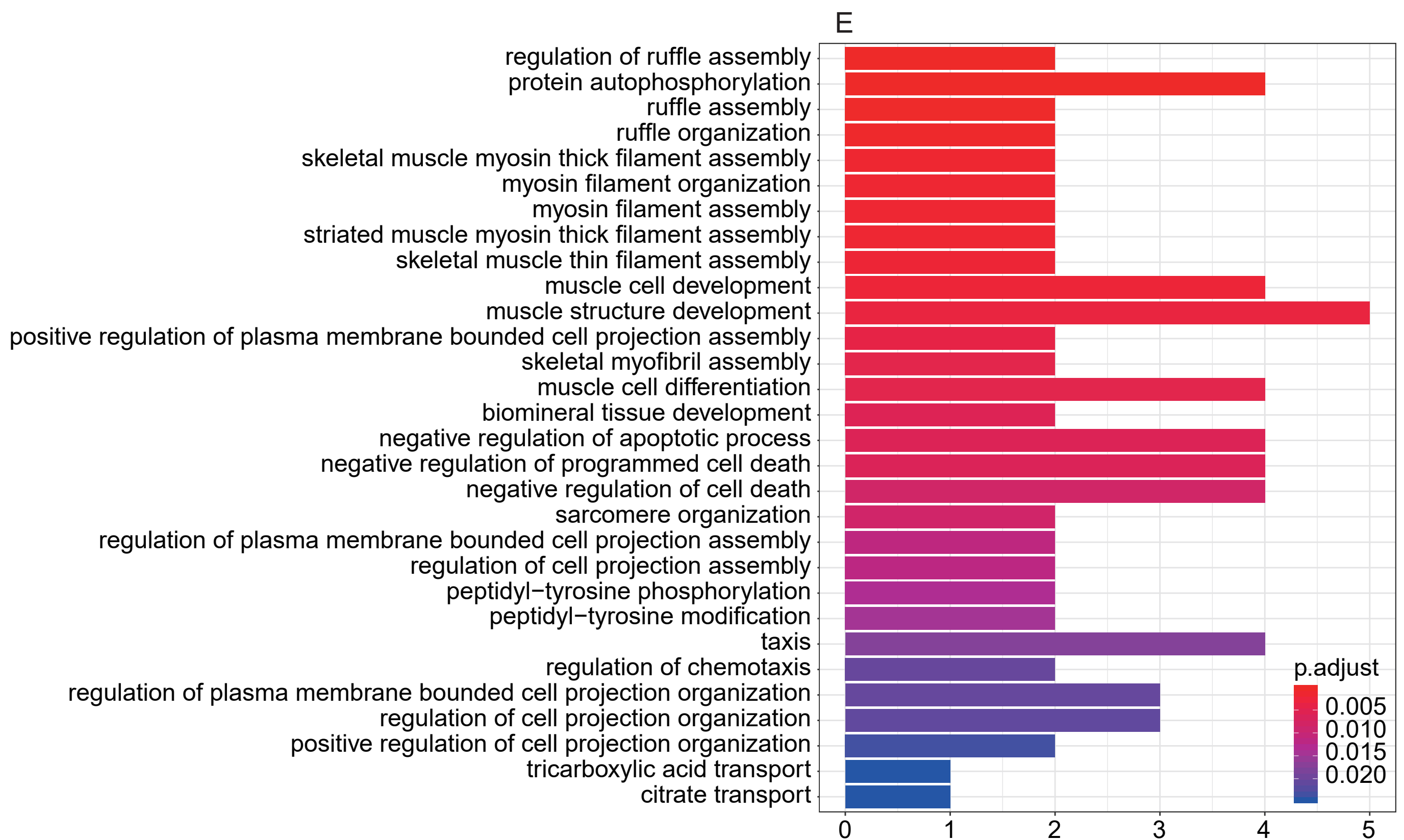

Supplement: Supplementary file 6 — Additional file 6. The gene expression profile in solvent control adult zebrafish group and EM-treated and solvent control adult male group. A. Comparison of differentially expressed gene in control male and female gonad. Volcano plot shows genes control female ovaries biased expression and control male testis biased expression. B-C. GO enrichment analysis based on genes which show sex-biased expression in control females and males. B. Female-biased gene GO enrichment analysis. C. Male-biased gene GO enrichment analysis. D. Comparison of differentially expressed gene in control male and EM-treated male gonad. Volcano plot shows genes control male testis biased expression and EM-treated male testis biased expression. E. GO enrichment analysis based on genes which show EM-treated male testis biased expression. The length of the bar corresponds to the number of genes enriched in the corresponding GO terms, and the color from red to blue represents the p.ajust value changes. [file 12864_2019_6437_MOESM6_ESM.pdf]
